# FROM CARE DYAD TO SUPPORT NETWORKS: PURCHASE INTENTION FOR HOME- AND COMMUNITY-BASED SERVICE OF CHINESE OLDER ADULTS

**DOI:** 10.1093/geroni/igae098.1147

**Published:** 2024-12-31

**Authors:** Zi Yan, Xin Sun

**Affiliations:** Waseda University, Tokyo, Japan; Fudan University, Shanghai, Shanghai, China (People’s Republic)

## Abstract

This study examines the underlying types of intergenerational solidarity and informal support networks among impaired older adults in China, and explains how they may influence the home- and community-based service purchase intention of these individuals. Based on the 2018 and 2020 waves of the Chinese Longitudinal Aging and Social Survey, a total of 3,564 older adults (aged 60 and above, with at least one child) were selected. First, we use K-means cluster analysis to identify four types of intergenerational solidarity among Chinese families: tight-knit, distant-but-intimate, independent-intimate, and emotional-detached. Secondly, we examined the size of three distinct types of informal support networks among Chinese older adults: general social networks, functional support networks, and emotional support networks. Subsequently, binary logistic regression was conducted to examine the relative impacts of intergenerational solidarity and informal support networks on impaired older adults’ purchase intention. Our findings highlighted the diverse impacts of informal functional/emotional support networks on older adults’ purchase intentions. These networks exhibit the potential to either substitute for or complement formal care, contingent upon the specific types of intergenerational solidarity. This research enriches the theoretical literature by incorporating both vertical and horizontal perspectives. It also enhances our understanding of the dynamics between ‘formal’ and ‘informal’ care within the Chinese context. This study underscores the necessity for precise targeting in service delivery, emphasizing the importance of considering the entire family and the influence of the older adults’ social network on service utilization and purchase decisions.

